# Spilling the Tea on HIV Prevention: Co‐Designing Sexual Health Education in Black‐Owned Baltimore Beauty Spaces

**DOI:** 10.1002/jia2.70221

**Published:** 2026-10-26

**Authors:** Sierra Cary‐Brown, Megan E. Mansfield, Meg Jordan, Stephen B. Thomas, Marie‐Claude Lavoie, Natalie Spicyn

**Affiliations:** ^1^ Institute of Human Virology University of Maryland School of Medicine Baltimore Maryland USA; ^2^ Department of Health Policy and Management University of Maryland College Park College Park Maryland USA

1

In the United States, Black women bear a disproportionate burden of new HIV acquisitions, accounting for 50% of HIV diagnoses but representing only 13% of the female population [1]. Similarly, in Baltimore, 19% of new HIV diagnoses in 2023 were among women [2]. Although pre‐exposure prophylaxis (PrEP) is readily available, uptake among women is low [3]. Multi‐level factors contribute to low PrEP uptake among Black women, including lack of awareness, medical distrust, gendered racism and stigma [4, 5]. New models of engagement are needed to better serve women, and we sought to explore novel methods for increasing PrEP awareness and engagement among Black women in Baltimore.

Beauty shops are intergenerational hubs for information exchange and peer support among Black women, making them trusted venues for community health promotion [6]. However, there is limited research examining the implementation of HIV prevention promotion in beauty shops. To address this gap, we recruited six Black Baltimore‐based beauty industry (hair and aesthetic) professionals to co‐design a community‐based HIV prevention intervention. These participants were identified through street outreach efforts and shop‐owner referral. Inclusion criteria were (1) being the shop owner‐operator, (2) having Black women comprise an estimated majority of clientele, and (3) shop location within Baltimore City or Baltimore County, Maryland. Although shop location cannot necessarily determine where clients reside, four of the six shops are in zip codes with a high HIV incidence rate [2], where 59.2% of the population identify as Black or African American. As of 2024, 36.1% of people in Baltimore had a Bachelor's degree or higher, the median household income was $62,177 and 58.9% of people were employed [7].

We used a five‐step human‐centred design [8] approach to co‐develop the intervention (Table 1).

**TABLE 1:** Summary of human‐centered design process activities and outputs.

Step	Activities	Outputs
**1. Empathize** understand personas’ needs, motivations, challenges	Creation and exploration of personas representing diverse experiences and community perspectives on HIV.	Two client personas generated to be used throughout the remaining steps.
**2. Define** identify personas’ highest priority problem and how to address it	Identify the challenges, pressures, passions and successes of the personas across their life‐course.	Identify core problem statements for which solutions need to be generated.
**3. Ideate** create multiple viable solutions for the problem	Iteration of various ideas for addressing problem statements.	Synthesis of ideas into an intervention—*Tea Talk*.
**4. Prototype** create first draft of the solution	Development of materials and processes for implementation.	Educational sessions Informational materials Procurement of resources
**5. Test** pilot the solution in the real world and collect feedback	Implementation of *Tea Talk* in participating shops (*n* = 6) for 3 months.	Website monitoring Regular check‐ins and reflections

This approach provided an empathetic lens to address root causes to challenges in HIV prevention among Black women in Baltimore. Steps 1–3 were completed through the course of four, 2‐h guided workshops conducted over Zoom. During these sessions, the participating beauty industry professionals (participants) were invited to create client personas that contextualized sexual health issues using a life‐course framework. During prototyping, we generated intervention materials and completed three 2‐h educational sessions in which medical and public health experts shared information on sexually transmitted infections (STI) treatment and prevention and gender inclusivity. Participants named the intervention *Tea Talk*, a hyperlocal community‐engaged intervention aiming to support HIV prevention by meeting Black women where they are both physically and culturally. *Tea Talk* included tea and snacks, attention‐grabbing posters with connections to local resources and pertinent educational materials. Critical components of the intervention included:

1. **Leveraging trusting relationship between beauty industry professionals and clients**. Beauty industry professionals build relationships with women in their communities through consistent, repeated interactions while providing beauty services that last hours. Beauty shops are non‐judgemental and comfortable, which participating professionals contrasted with typical healthcare settings. As a result, women speak openly about relationships and sex, offering participants unique opportunities for culturally responsive conversations about sexual health and HIV prevention with inherent sensitivity to racial and gender stigma.
2. **Educational training for participating beauty industry professionals**. Acknowledging a lack of formal sexual health education beyond secondary school, participants requested in‐depth educational and role‐playing scenario training on sex, gender, sexuality and biomedical prevention tools. They expressed a desire to bolster their confidence as lay educators and trusted sources of information, while avoiding the inadvertent spread of misinformation.
3. **Use of visibly interesting yet discreet materials**. Intervention materials were designed with bright colours and intriguing text to spark conversation. When clients asked about the materials (e.g. “what's the tea?”), the participating beauty industry professionals would offer tailored messaging about HIV prevention. Throughout the design process, participants highlighted concerns about HIV stigma; Accordingly, they requested materials not explicitly mention “HIV” or “PrEP” due to fears of deterring clients.

*Tea Talk* was piloted (step 5) in six shops for 3 months (March−June 2025). During testing, participating beauty industry professionals met every 2 weeks in a community of practice to discuss their experiences implementing the programme. Learnings included the impact of different business structures (e.g. single owner vs. multiple partners) as well as clientele age impacting engagement. We used shop‐specific QR codes to monitor client access to the *Tea Talk* PrEP informational website, which was accessed by clientele a total of 62 times during the pilot period. Participants reported strong client interest in sharing educational and preventative materials with others. After the 3‐month pilot, participants completed an interview to discuss their experiences with the co‐design process, implementation, and suggestions for changes and future directions. Recommendations included incorporation of digital materials and adding mental health components to the intervention.

Overall, the co‐design process was enthusiastically received by the beauty industry participants, who indicated that they found *Tea Talk* feasible to implement and broadly acceptable to their clientele. Primary insights included:

1. **The co‐design process was instrumental in shifting perceptions of who is “at risk” for HIV**. Existing research suggests that one potential reason for the disparity in new HIV acquisitions among Black women is low perceived need of PrEP [9]. Beauty industry participants shared a shift in their perception of PrEP from it being for gay men to it also benefiting Black women. Notably, participants stressed framing HIV prevention as a community wellness endeavour.
2. **Beauty industry professionals are motivated to be community‐based sexual‐health educators**. Beauty industry participants reported that engaging in the co‐design process and implementing the intervention aligned with their overarching ethic of promoting wellness in their communities. Participants reported high civic engagement and identifying as community resources, which also motivated their participation.
3. **Co‐design enables the development of contextually appropriate tools**. Our experiences underscore the importance of using inclusive design methods to support development and implementation of public health interventions. Such approaches promote thoughtful language and context‐appropriate framing to ensure acceptability and clarity of messaging. For *Tea Talk*, participants encouraged removal of explicit references to HIV and PrEP. This discretion facilitated conversation, which better leveraged the existing trusting relationships between beauty industry professionals and their clients.

The development of *Tea Talk* demonstrates the value of human‐centered design methods in fostering ownership and ensuring relevance for community‐based health initiatives. Further, *Tea Talk* demonstrates a potential model for reaching structurally underserved populations. Community‐based outreach offers a critical advantage over clinic‐based models by delivering information about new PrEP formulations directly within environments where trust is already established, reducing the structural and social barriers that have historically limited PrEP uptake among Black women. Rather than expecting individuals to overcome barriers to care, *Tea Talk* shifts the focus to meeting people in their own space. In doing so, this approach respects community leaders as not only partners but experts, co‐creators, and supporters of health interventions in their communities. As HIV prevention programmes seek to expand their reach, embedding co‐designed interventions in community‐trusted environments may be critical to ensuring equitable access and sustainability of these vital health services.

## Author Contributions

**Sierra Cary‐Brown**: conceptualization, data curation, investigation, formal analysis, funding acquisition, project administration, writing – original draft, writing – review and editing. **Megan E. Mansfield**: methodology, data curation, investigation, formal analysis, visualization, writing – original draft, writing – review and editing. **Meg Jordan**: methodology, data curation, project administration, writing – original draft. **Stephen B. Thomas**: conceptualization, methodology, supervision, funding acquisition, writing – original draft, writing – rewview and editing. **Marie‐Claude Lavoie**: methodology, writing – review and editing. **Natalie Spicyn**: conceptualization, methodology, investigation, validation, supervision, funding acquisition, project administration, writing – review and editing.

## Funding

This research was supported in part by the shared resources of the University of Maryland, Baltimore (UMB) Institute for Clinical & Translational Research (ICTR) through funding from the National Center for Advancing Translational Sciences (NCATS) of the National Institutes of Health (NIH) under award number UM1TR004926. The content is solely the responsibility of the authors and does not necessarily represent the official views of the National Institutes of Health.

## Conflicts of Interest

The authors declare that they have no conflicts of interest.

## Data Availability

The data that support the findings of this study are available on request from the corresponding author. The data are not publicly available due to privacy or ethical restrictions.
